# The Origin and Diversification of the Hyperdiverse Flora in the Chocó Biogeographic Region

**DOI:** 10.3389/fpls.2019.01328

**Published:** 2019-12-06

**Authors:** Oscar Alejandro Pérez-Escobar, Eve Lucas, Carlos Jaramillo, Alexandre Monro, Sarah K. Morris, Diego Bogarín, Deborah Greer, Steven Dodsworth, José Aguilar-Cano, Andrea Sanchez Meseguer, Alexandre Antonelli

**Affiliations:** ^1^Comparative Plant and Fungal Biology Department, Royal Botanic Gardens Kew, London, United Kingdom; ^2^Gothenburg Global Biodiversity Centre, Gothenburg, Sweden; ^3^Smithsonian Tropical Research Institute, Panama, Panama; ^4^ISEM, U. Montpellier, CNRS, EPHE, IRD, Montpellier, France; ^5^Universidad de Costa Rica, Jardín Botánico Lankester, Cartago, Costa Rica; ^6^Department of Environment, Food and Rural Affairs, London, United Kingdom; ^7^School of Life Sciences, University of Bedfordshire, Luton, United Kingdom; ^8^Research Institute Alexander von Humboldt, Bogota, Colombia; ^9^Real Jardín Botánico de Madrid (RJB-CSIC), Madrid, Spain

**Keywords:** biogeography, macroevolution, neotropical region, hyper-diversity, Andean uplift, Chocó, Central America

## Abstract

Extremely high levels of plant diversity in the American tropics are derived from multiple interactions between biotic and abiotic factors. Previous studies have focused on macro-evolutionary dynamics of the Tropical Andes, Amazonia, and Brazil’s Cerrado and Atlantic forests during the last decade. Yet, other equally important Neotropical biodiversity hotspots have been severely neglected. This is particularly true for the Chocó region on the north-western coast of South and Central America. This geologically complex region is Earth’s ninth most biodiverse hotspot, hosting approximately 3% of all known plant species. Here, we test Gentry’s [1982a,b] hypothesis of a northern Andean-Central American Pleistocene origin of the Chocoan flora using phylogenetic reconstructions of representative plant lineages in the American tropics. We show that plant diversity in the Chocó is derived mostly from Andean immigrants. Contributions from more distant biogeographical areas also exist but are fewer. We also identify a strong floristic connection between the Chocó and Central America, revealed by multiple migrations into the Chocó during the last 5 Ma. The dated phylogenetic reconstructions suggest a Plio-Pleistocene onset of the extant Chocó flora. Taken together, these results support to a limited extend Gentry’s hypothesis of a Pleistocene origin and of a compound assembly of the Chocoan biodiversity hotspot. Strong Central American–Chocoan floristic affinity may be partly explained by the accretion of a land mass derived from the Caribbean plate to north-western South America. Additional densely sampled phylogenies of Chocoan lineages also well represented across the Neotropics could enlighten the role of land mass movements through time in the assembly of floras in Neotropical biodiversity hotspots.

## Introduction

“Geologically the Chocó represents a recent emergence formed as a part of the main Andean uplift, perhaps only in the mid-Pleistocene” (Gentry, 1982b).

The American tropics (a.k.a. the Neotropical realm) extends from central Mexico to southern South America including the Caribbean (Antonelli et al., 2018a) and are home to six of the most species-rich biodiversity hotspots on Earth (Mittermeier et al., 2011). The origin of plant Neotropical megadiversity and the processes driving this diversification have been studied over the past four decades (e.g. Gentry, 1982b; Gentry, 1992; Antonelli et al., 2009, Antonelli et al., 2018b; Hoorn et al., 2010). As a result, several biotic (plant–organism interactions) and abiotic factors (e.g. climate, orogeny, and plant migration dynamics) have been posited to have influenced the diversification of plant lineages in the region (Hughes and Eastwood, 2006; Antonelli and Sanmartín, 2011a; Uribe-Convers and Tank, 2015; Lagomarsino et al., 2016; Pérez-Escobar et al., 2017a).

Historically, phylogenetic-based studies have heavily focused on a restricted subset of biogeographical regions or biodiversity hotspots within the American tropics (e.g. Amazonia, tropical Andes, Central America, Brazil’s Cerrado and Atlantic forests, and Seasonally Dry Forests) and their most prominent plant groups, including Annonaceae (Erkens et al., 2007; Pirie et al., 2018), Arecaceae (Bacon et al., 2013; Cano et al., 2018), Campanulaceae (Lagomarsino et al., 2016), Fabaceae (Richardson et al., 2001; Hughes and Eastwood, 2006; Nevado et al., 2016; Schley et al., 2018), Myrtaceae (Vasconcelos et al., 2019), and Orchidaceae (Martins et al., 2018; Pérez-Escobar et al., 2017a, Pérez-Escobar et al., 2017c). This is likely due to the possibilities of producing relatively well sampled phylogenies for such groups, built upon decades of plant collection and documentation in biogeographical regions that are relatively easy to access (Eiserhardt et al., 2017).

## Biogeography, Climate and Diversity of the Chocó

Perhaps one of the least understood biodiversity hotspots in terms of species diversity and evolution in the American tropics is the Chocó biogeographic region (A.K.A. Tumbes-Chocó-Magdalena, henceforth referred as the “Chocó”; Cano et al., 2017). The Chocó is the world’s ninth most biodiverse hotspot and hosts nearly 3% (∼11,000 species) of all plant species (Christenhusz et al., 2017), including ∼2,750 endemic species, in less than 0.2% of the Earth’s land surface (Gentry, 1982b; Myers et al., 2000; Mittermeier et al., 2011). Thus, the Chocoan landscape is as rich as other megadiverse, but considerably larger biogeographical regions such as Central America. Yet, substantial knowledge gaps in the mode and tempo of evolution of the Chocoan flora still exist. These mainly stem from the notably limited availability of comprehensively sampled phylogenies of plant groups prominent in the region but also distributed across the American continent (Jaramillo, 2006). To our knowledge, no study has yet specifically attempted to disentangle the origin and drivers of diversification of the hyper diverse flora of this region using phylogenetic frameworks. Those who have sampled plant Chocoan diversity often include, at most, a handful of species that are distributed and/or restricted to the Chocó (e.g. Pérez-Escobar, 2016; Pirie et al., 2018; Canal et al., 2019; Thode et al., 2019).

**Figure 1:**
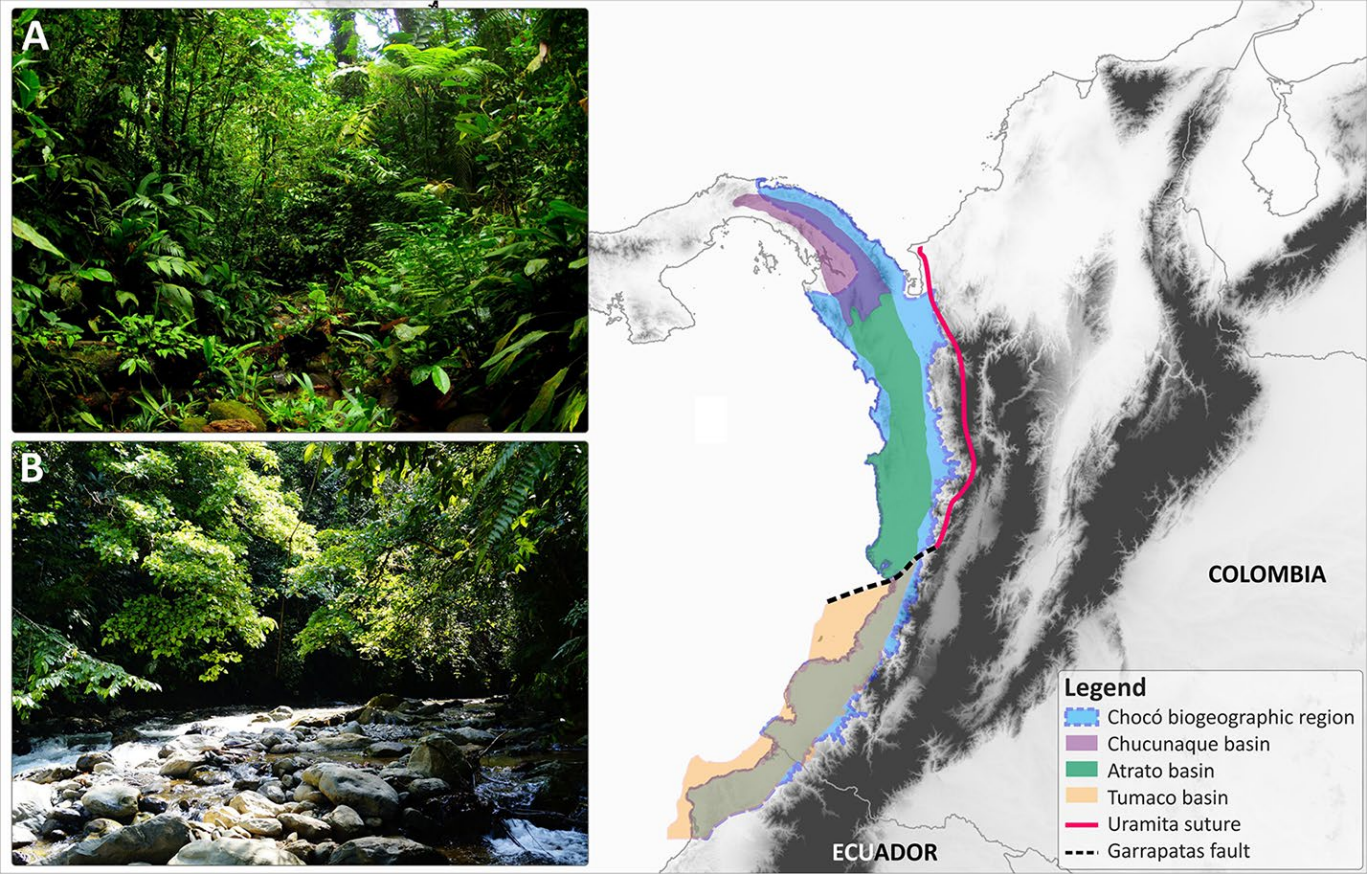
Geographical extent of the Chocó biogeographic region (highlighted in blue) in Central and South America as defined by Myers (2000) and its compound geological origin. The geological extensions of the Chucunaque, Atrato, and Tumaco basins are indicated with colour coded polygons. The location of the Uramita suture and the Garrapatas fault are also provided. **(A)** Lowland wet forest in north-western Colombia in Purricha (Chocó Department); **(B)** Pre-montane gallery forest in central-western Colombia, near Queremal town (Valle del Cauca department). Photos: R. Cámara-Leret and O. Pérez]

Phytosociological communities in the Chocó broadly occur in 20 ecosystems types, including lowland wet forests (the dominant ecosystem), forest swamps, grasslands, coastal mangroves, and montane cloud forests (Rangel-Chui, 2011; Cano et al., 2017). The precise northern extent and delimitation of the Chocó into the Darien Gap remain contentious. Different delimitations have been employed in biogeographical studies (e.g. Gentry, 1986; Morrone, 2006; Antonelli et al., 2009; Rangel-Chui, 2011; Pérez-Escobar et al., 2017b; Cano et al., 2018), but most of these encompass the area between Central Panama (the geographical limit between Colombia and Panama) or from Southern Nicaragua to north-western Venezuela (Morrone, 2006). The southern fringes are less debated, usually marked by the wet forests of the Pacific coast of northern Ecuador (Esmeraldas Province; **Figure 1**).

A particular aspect of the Chocó is its extreme precipitation and humidity, which renders this region one of the “wettest places on earth” (Gentry, 1986). With a recorded annual precipitation of 3,000–11,700 mm, the Chocó stands as the second rainiest place after Cherrapunjee (India), which receives an annual mean precipitation of 12,000 mm (Banerjee et al., 2012). Detailed floristic studies led by Gentry (1986) in 0.1 ha plots in areas with different levels of rainfall in the Chocó revealed differences in species diversity correlated with precipitation. A strikingly similar correlation between species richness, individual abundancy and precipitation was found more recently to occur in palms (Copete et al., 2019). More species have been recorded from plots located in wetter areas of the Chocó than in plots situated in drier localities, even though all sites present higher individual densities compared with most other continental ecosystems around the world (Gentry, 1986).

## The Intricate Climatic and Geological History of Central America and Its Influence on the Origin and Diversification of the Chocoan Flora

The geological formation of the Chocó entailed very complex processes, but is mostly the by-product of interactions between the Nazca, Cocos, and South American plates during the past 100 million years (see Borrero et al., 2012; Montes et al., 2015; Cardona et al., 2018; León et al., 2018 for extensive reviews on the tectonic evolution of the region). The Chocó is composed of two geological blocks, the Chucunaque–Atrato to the north and the Tumaco to the south (**Figure 1**). The Chucunaque–Atrato block belongs to the Panama microplate, which is a piece of the trailing edge of the Caribbean plate. It is an intra-arc sedimentary basin bounded by magmatic arcs, which extends from central Panama to the middle part of the Chocó region (Duque-Caro, 1990a; Duque-Caro, 1990b; Coates et al., 2004; Wegner et al., 2011; **Figure 1**). In other words, the geological origin of the Chocó is Central American rather than South American.

The collision of the Chucunaque–Atrato basin and its associated magmatic arc with South America commenced during the late Miocene. By ∼10 Ma, the collision had fused the Panamanian magmatic arc with the western Andes of Colombia along the Uramita suture (Montes et al., 2015; León et al., 2018). Paleogeographic reconstructions indicate that at 10 Ma, most of the Chucunaque–Atrato basin comprised shallow marine environments. Thus, by the late Miocene, in the region currently occupied by the Chocó land existed only in a) the Panamanian magmatic eastern arc that fused together with the western Andes along the Uramite suture and b) in the western arc (i.e. the Baudo range; Coates et al., 2004; Jaramillo, 2018).

A second developmental phase of the Chocó began following the onset of the collision of South America with the trailing edge of the Caribbean plate (the Panama microplate) prompting a progressive shallowing of the Chucunaque–Atrato basin, which transformed marine settings into terrestrial landscapes. The collision appears to have segmented the basin into two blocks, Chucunaque in Panama and Atrato in Colombia (**Figure 1**). In the Atrato basin, this shallowing probably moved from south to north, with fully terrestrial environments no older than 3.1 Ma (Duque-Caro, 1990a, Duque-Caro, 1990b), although the precise dating of the onset is still unknown and could be much younger. The southern boundary of the Atrato basin is bounded by the Garrapatas fault (**Figure 1**). South of Garrapatas lies the Tumaco forearc basin (Borrero et al., 2012), which covers the southern segment of the Chocó. The Tumaco forearc basin formed in an island arc setting (Borrero et al., 2012) and seems also to be part of the Caribbean plate, having collided earlier with South America compared to the Chucunaque-Atrato basin, although additional studies are needed to confirm the time of collision. Fully terrestrial environments are no older than 4.2 Ma (Borrero et al., 2012) but more precise dating of the onset of terrestrial landscape is still required as it could be younger.

Modern rainfall patterns in the Chocó are amongst the highest in the world and are driven by the Chocó jet stream centred around 5° N (Poveda and Mesa, 2000). The strength of the stream is related to temperature surface gradients between western Colombia and the tropical east Pacific (El Niño 1 + 2 geographical regions) with precipitation over the Chocó related to the high elevation of the western Cordillera, which acts as a barrier to the jet stream. The temperature gradient along the tropical Pacific intensified with the onset of the Pleistocene (Fedorov et al., 2013). Therefore, the origin of the modern Chocó jet stream may be linked with the onset of the Pleistocene.

Taken together, the geological history of the Chocó suggests that the modern landscape (i.e. fully terrestrial with high rates of precipitation) was only established by the early Pleistocene (i.e. ∼2.7 Ma). The closest source areas of plant migrants to populate this newly developed terrestrial set of ecosystems could have been the lowlands of Panama to the north, and the montane habitats of the western Cordillera to the east. In contrast, the region south of Chocó highly increased its aridity during the late Neogene, thus being an unlikely source of Chocoan plant lineages.

## Hypotheses on the Mode and Tempo of Evolution of the Chocoan Flora

One peculiarity of the floristic composition of the Chocó is its high endemism that appears restricted to species, as there is only one endemic genus in the region (the recently described *Sabinaria* in the Arecaceae; Bernal and Galeano, 2013). To date, no endemic plant families have been found. Such curious patterns of endemism have attracted the attention of botanists for decades (Gentry, 1982b; Forero and Gentry, 1989; Galeano et al., 1998). Gentry’s seminal floristic work (1986) and the geological evidence available already nearly 50 years ago (Haffer, 1970), led him (Gentry, 1982a) to conclude that: a) extremely high levels of rainfall in the Chocó are linked to its rich plant diversity (Jaramillo, 2006) and its maintenance (Behling et al., 1998): b) Chocoan plant lineages may have derived from the Northern Andean region with possible contributions from Central America; c) the geological origin of the Chocó dates back to the middle Pleistocene and was part of the mountain building processes of the Colombian Andes. Thus, the floristic assembly of the Chocó, which benefitted from the biotic exchange between the actively rising Northern Andes and Central America, was supposedly formed Chocó from around 1 Mya onwards.

To test the scenarios postulated by Gentry, phylogenetic comparative methods based on densely sampled and representative phylogenies could reveal the age, direction and frequency of biotic exchanges between the Chocó and other regions (Pennington et al., 2004). In addition, they could inform on the role of geographical events that have shaped the diversification of the Chocoan flora (Winterton et al., 2014). A similar approach has been used to investigate the evolution of the savanna-adapted lineages in the South American Cerrado, which for plants is also very rich in species but is similarly depauperate at higher taxonomic levels of genera and families (Simon et al., 2009).

**Figure 2:**
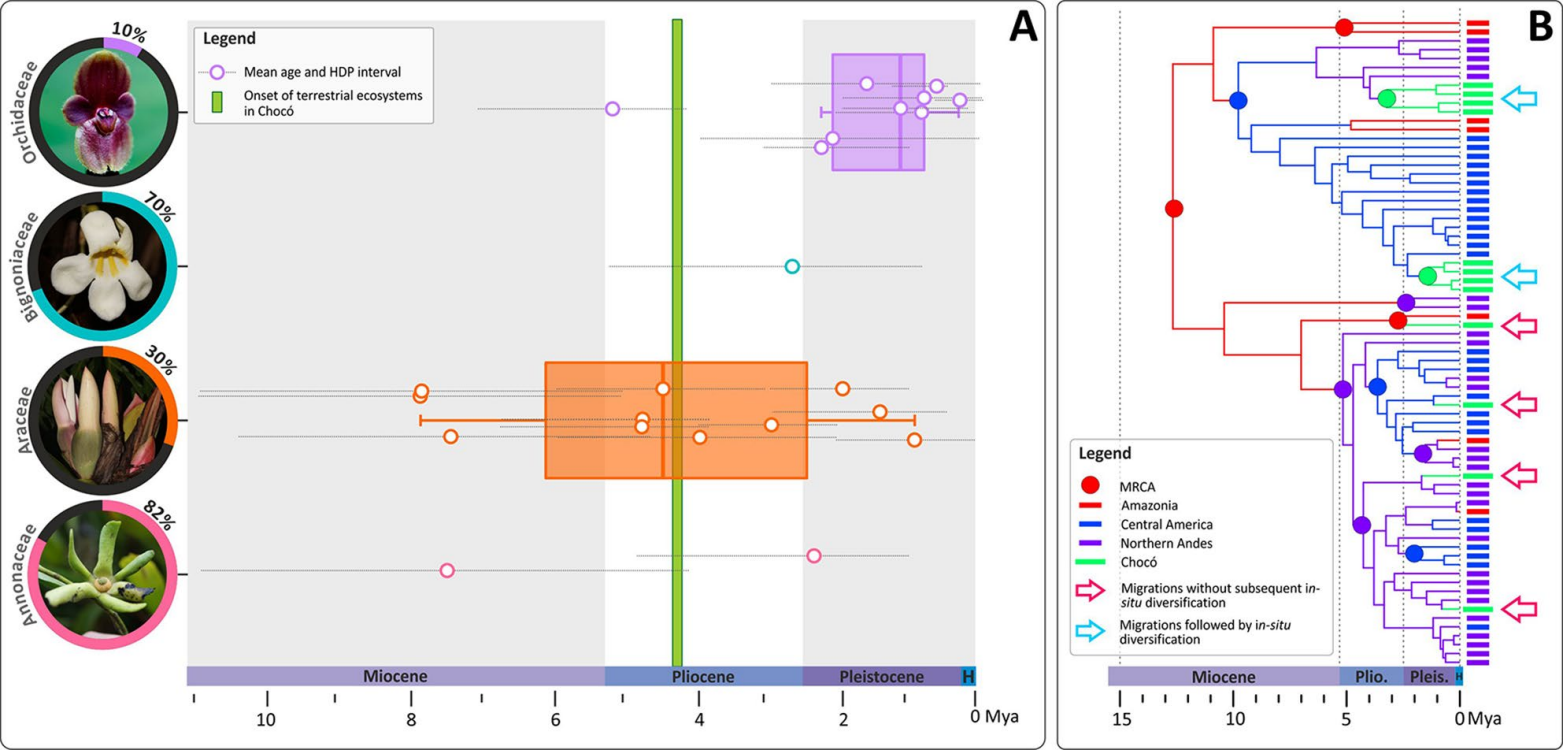
Temporal migration dynamics of selected Neotropical plant lineages represented in the Chocó and their diversification patterns. **(A)** Mean ages and their corresponding 95% High Density Probabilities (HDP) of Most Recent Common Ancestors (MRCA) of plant species/clades distributed in the Chocó. Ages estimates are colour coded by family and were obtained from published phylogenies of Cymbidieae and Pleurothallidinae (Orchidaceae: Pérez-Escobar et al., 2017a), *Amphilophium* (Bignoniaceae: Thode et al., 2019), *Philodendron* (Araceae: Canal et al., 2019), *Cremastosperma* and *Mosannona* (Annonaceae: Pirie et al., 2018). Boxplots representing mean values and quartiles were computed whenever four or more observations per plant family were available. [Inset: The proportion of species sampled vs the total known of species diversity in each surveyed phylogeny is provided with a picture of the corresponding plant family (Orchidaceae: *Pleurothallis pierryi*; Bignoniaceae: *Anemopaegma orbiculatum*; Araceae: *Philodendron* sp.; Annonaceae: *Cremastosperma* sp.)]. The green bar represents the approximate age of the terrestrial ecosystem onset in the Chocó. **(B)** A schematic phylogeny representing the two most prominent diversification patterns of Chocoan lineages, all of which appear to have occurred during the last ∼8 Ma: 1) migration from adjacent or distant biogeographical areas into the Chocó followed by *in-situ* diversification (blue arrows); 2) migration into the Chocó without subsequent diversification. Photos: O. Pérez.

Few robust phylogenies for Chocoan plant species exist, and virtually all of them have sparse regional taxon sampling. Those available include Annonaceae [*Cremastosperma* (29 species/82% sampled - one Chocoan species) and *Mosannona* (14 species/78% sampled — one Chocoan species); Pirie et al., 2018], Bignoniaceae [*Amphilophium* (47 species/70% sampled — one Chocoan species); Thode et al., 2019], the two most species-rich groups of Neotropical orchids [tribe Cymbidieae (∼3,300 species/26% sampled — five Chocoan species) and subtribe Pleurothallidinae (∼5,100 species/11% sampled – four Chocoan species); Pérez-Escobar et al., 2017a] and *Philodendron* (Araceae, 560 species/30% sampled — 16 Chocoan species; Canal et al., 2019). These studies provide an excellent opportunity to test Gentry’s ideas of a Pleistocene Northern Andean–Central American origin for the Chocó flora as they include different levels of sampling (i.e. 10% to ∼80% of the known species diversity of each clade; the exact proportion of species diversity sampled is provided in **Figure 2**).

Ancestral area estimations in Cymbidieae and Pleurothallidinae (**Figures S1A, B**–**S2A and B**) revealed eleven migrations to the Chocó, which could be classified into two patterns: a) Northern Andes to Chocó and b) Central America to Chocó. The first migration pattern included four dispersals from the late Miocene (∼6 Mya) to as recently as 2 Mya (**Figures S1B** and **S2B**). The second pattern consisted of four migrations from the adjacent Central America region towards the Chocó. Notably, most of these migrations occurred recently (∼2 Mya) and no recolonizations from the Chocó back to the Northern Andes or Central America were detected (**Figures S1B** and **S2B**). Ancestral character estimations of altitude as a continuous character indicated that most of the Northern Andean migrants were originally adapted to montane elevations, between elevations of ∼1,200 to 1,700 m. In contrast, most Central American migrants were probably first adapted to lowland environments. Descendent branches from Northern Andean migrants subsequently adapted to pre-montane environments (i.e. elevations below ∼500 m; **Figures S1C** and **S2C**). The putative montane-to-lowland adaptations into the Chocoan premontane environments revealed by our ACE analyses are striking, reflecting results reported for Chloranthaceae (*Hedyosmum scaberrimum*) and Magnoliaceae (e.g. *Magnolia calimaensis*) in the Chocó (Gentry, 1986; Galeano et al., 1998; Antonelli and Sanmartín, 2011b). Nevertheless, understanding how frequent the out-of-the-mountains migration pattern has been across Chocoan orchid lineages deserves further investigation.

Species level phylogenies of *Cremastosperma, Mosannona*, and *Philodendron* revealed similar modes and tempos of migration into the Chocó (**Figure 2**). Lineage colonizations occurred mostly after divergences from Central American and Andean ancestors between ∼7 and 1 Ma. Less frequent, alternative migration routes were recovered in *Amphilophium* and in the predominantly lowland epiphytic orchid *Cycnoches* (Pérez-Escobar et al., 2017b; Pérez-Escobar et al., 2017c). These are lineages from far-away biogeographical regions such as Amazonia which are, or have been, isolated from the Chocó by major geographical barriers (e.g. the Northern Andes). Here, a single migration from Amazonia towards the Chocó took place ∼1 Mya in *Cycnoches* (Pérez-Escobar et al., 2017c), whilst an older colonization (∼3 Ma) also from Amazonia occurred in *Amphilophium* (Thode et al., 2019).

The most common pattern of diversification across all surveyed lineages is that of colonization of the Chocó without subsequent *in-situ* speciation. Of the studies considered here, only two cases of migration followed by *in-situ* diversification were recovered, one in *Anthurium* (∼ 5 Mya, three species; Canal et al., 2019) and one in *Cycnoches* (∼1 Mya, two species; Pérez-Escobar, 2016). This apparent lack of *in-situ* plant diversification in the region might be explained by the very recent Pleistocene setting of the Chocoan modern landscape or alternatively, by the incomplete sampling of these phylogenies. However, the stark contrast between the high levels of species endemism and very limited number of genera restricted to the Chocó reflects the limited morphological divergence among lineages diversifying in the Chocó. Migrations without subsequent diversification is a remarkable pattern that is unusual in biodiversity hotspots [e.g. Tropical Andes, Seasonally Dry Forests and Indo-Pacific coral triangle (Pennington et al., 2009; DRYFLOR, 2016; Huang et al., 2017; Pérez-Escobar et al., 2018; Testo et al., 2019; Vasconcelos et al., 2019)] and deserves further study, for instance to investigate the possibility of cryptic species and on-going speciation. Additional phylogenies with representative sampling at species and population level in groups with disparate life histories and diversification rates could reveal how frequent *in-situ* diversification is and it is restricted to rapidly diversifying groups or not.

## A Compound Origin of the Chocoan Flora

Phylogenetic comparative analyses of early Miocene lineages support Gentry’s idea of a Northern Andean–Central American origin of the Chocoan flora (Pérez-Escobar et al., 2017a). The proximity of the Northern Andean and Central American biogeographical regions to the Chocó could explain the mixed origin of the Chocó flora. However, phylogenetic frameworks of plant clades with a Pliocene age suggest that contributions from more distant biogeographical areas are also relevant for the assembly of the Chocó flora (Pérez-Escobar et al., 2017c). More recently, an example of a palm species with a cross-Chocoan distribution range and constant gene flow (Escobar et al., 2018) also support this migration pattern, indicating that the Chocó is a permeable, accessible region, not totally isolated from either adjacent or more distant biogeographical areas.

Late Miocene and Pliocene migrations into the Chocó might at first seem unlikely given that the modern lowland landscape of the Chocó region is not older than ∼3 Ma. However, the foothills of the Baudo in west Chocó and western Cordillera (west of the Uramita suture), also considered to be part of the Chocó region, are terrestrial landscapes established already by 6 Ma. As such, they could have served as a host area for plant migrants since the late Miocene. Thus, the timing of migrations into the Chocó, irrespective of the source biogeographical area, provides strong support for a predominantly Pleistocene geological onset of the region in South America, hence largely supporting Gentry’s assumption of a Pleistocene origin of the Chocó.

Taken together, these spatio-temporal dynamics suggest that a) allopatric speciation in plants promoted by the Andean uplift seems unlikely to have played a role in the formation of the Chocoan flora given the very recent age of migrations from biogeographical regions isolated by the Northern Andes and the older age (∼10 Ma) of formation of montane–alpine environments in the Northern part of the Cordillera (Hoorn et al., 2010; Hoorn and Wesselingh, 2010); b) Certain elements of Chocoan plant diversity have an earlier origin than Gentry thought, as evidenced by the early Pliocene migrations from the Northern Andes to the Chocó accounted in Pleurothallidinae; c) there has been strong biotic exchange between Central America and Chocó, particularly evident during the Pleistocene and seemingly unlinked with the timing of the closure of the Central American Seaway.

The current proximity of Central America to Chocó and/or the ability of orchid seeds to remain airborne for prolonged periods (Arditti and Ghani, 2000) and thus cover huge distances (Murren and Ellison, 1998) could explain the high frequency of biotic exchange between Central America and the Chocó for this family. The timing of the onset of modern Chocoan landscapes in turn could account for the similar age of these migrations (ranging from ∼5–2 Mya). Moreover, biotic contributions from other biogeographical areas like Amazonia also took place, suggesting that the Chocoan flora could be derived as well, but to a lesser extent, from other regions than just the Northern Andes and Central America.

## Geological History and Floristic Similarities of Central America and the Chocó Block

The floristic affinities of the Chocó and Central America have puzzled botanists for decades. Gentry’s notion of Central American contributions to the origin of the Chocoan flora was based on his observations of the striking resemblance of the species diversity shared between both regions in key family groups, including Annonaceae and Rubiaceae (Gentry, 1982b; Gentry, 1986). The composition of the orchid diversity in Central America mirrors in part that of the Chocó. Particularly, groups from the subtribe Zygopetalinae (e.g. *Houlletia*, *Huntleya*) and Maxillariinae (e.g. *Camaridium*) that peak in species-richness in the lowland and premontane regions of Central America, are also well represented in the Chocó (Urreta, 2005; Bogarín et al., 2015; Kirby, 2016). Comparative floristic studies focused on selected Araceae genera and pteridophytes in general (Lellinger, 1975) are in line with these findings (Mora et al., 2006), and an older idea of a more expansive Chocó region that extended to the Nicaraguan–Costa Rican border has received some support (Lellinger and de la Sota, 1978). However, distribution patterns of palms revealed that the Darien Gap on the Panama–Colombian border is an effective dispersal barrier for bidirectional exchange between South and Central America, at least for lineages that are dependent on other organisms for dispersal (Cano et al., 2017).

The Central American origin of one part of the Chocó region might provide a more plausible explanation for the strong floristic affinity between Central America and the Chocó. Here, the few chronograms of plant lineages available provide evidence of floristic admixture between Central and South America triggered by the accretion of the Chocó block. Comparable admixture has been suggested for other merging landmasses, such as the Indian subcontinent colliding with Asia (Thornhill et al., 2015). Intermingled clades of *Dussia* in the Leguminosae distributed in Central America and the Chocó suggest a nearly simultaneous migration from Central America into the Chocó, with concomitant diversification towards the late Miocene (Winterton et al., 2014). A similar late Pliocene migration pattern has been reported in *Cremastosperma* (Annonaceae), where a Central American common ancestor appears to have given rise to an endemic Central American and Chocoan species pair (*C. westrae* and *C. novogranatense*, respectively; Pirie et al., 2018). However, whether similar diversification patterns occur in other prominent plant lineages with more localized modes of dispersal remains to be tested.

## New Research Questions and Future Directions

Our detailed evaluation of migrations-through-time of selected Neotropical plant lineages between Neotropical regions and the Chocó reveals that the assembly of the Chocó flora is more complex than first proposed by Gentry. Whilst the timing of the onset of modern Chocoan landscapes could explain the poor endemism of genera and families in the Chocó, the influence of other biotic and abiotic variables in the diversification of the Chocoan flora remains unclear and begs further investigation. These include evaluating a potential role of extinction (within and between clades, and in relation to landscape changes) in shaping the endemicity of the Chocó region and understanding the influence of historical changes in humidity and precipitation on speciation. Moreover, future studies should focus on understanding the diversity and affinity of poorly studied but prominent plant lineages of the Central American floras, to facilitate comparison of floristic affinities and biotic interchange. Detailed floristic studies in the northern and southern fringes of the Chocó could further clarify the delimitation of the Chocó biogeographic region by comparing similarities in distribution patterns across plant groups (Vilhena and Antonelli, 2015).

Solid phylogenomic frameworks coupled with reliable distribution data of plant groups well represented in Central America and the Chocó (such as Araceae, Fabaceae, Myrtaceae, Melastomataceae, Moraceae, Rubiaceae, and Urticaceae) should help tease apart the role of modes of dispersal, plant habit, or life-form in determining the mode and tempo of plant migrations into the Chocó. For example, biogeographical studies of densely species level phylogenies could distinguish the roles of montane areas in the Northern Andean cordilleras and the Panamanian magmatic arcs (i.e. the western Cordillera in South America) as source of migrants into the Chocó lowlands. Furthermore, it would be particularly interesting to investigate in more detail the impact of the accretion of the Chocó Block to the South American plate. That event must not only have facilitated plant dispersal (Bacon et al., 2015) but also led to important climatic and edaphic changes in the Chocó. The direction and frequency of migrations across multiple plant lineages, coupled with changes in diversification rates and their relation to environmental changes, would shed further light onto the assembly of the Chocoan flora and its dynamics.

## Author Contributions

OP-E, SM, EL, AM, and AA designed the research. OP-E, CJ, EL, SM, AA, DB, DG, SD, JA-C, ASM, and AA performed the research. OP-E, CJ, EL, AM, and AA wrote the paper with contributions from all authors.

## Funding

OP-E is supported by the Lady Sainsbury Orchid Fellowship at the Royal Botanic Gardens, Kew.

## Conflict of Interest

The authors declare that the research was conducted in the absence of any commercial or financial relationships that could be construed as a potential conflict of interest.

## Supplementary Material

Figure S1 Spatio-temporal macro-evolutionary dynamics of the orchid clade Pleurothallidinae in the American tropics (adapted from Pérez-Escobar et al., 2017a). (A) Bayesian chronogram of the Pleurothallidinae, with ancestral areas estimated at nodes using the Dispersal, Extinction and Cladogenesis and the founder speciation event models as implemented in the R package BioGeoBEARS. Branches are colour coded according to the ancestral area estimated at the parent node. The distribution of the species sampled in the phylogeny is shown as horizontal colour coded bars in front of the tree terminals. Four clades with nested migration events to the Chocó region are highlighted in grey and labelled with roman numerals (I–IV). (B). Zoom-in on clades with nested migrations to the Chocó region (highlighted with pink arrows) (Inset: a: *Anathallis funerea*, b: *Anathallis lewisiae*, c: *Dracula* sp., d: *Specklinia pereziana*, e: *Specklinia berolinensis*). Letters at nodes represent inferred ancestral areas. (C) Maximum Likelihood Ancestral State Estimation analyses of altitude as a continuous variable of clades I-IV using a Brownian motion model as implemented in the R package phytools. Branches are colour coded as a function of estimated ancestral altitude values. [Inset: mean paleo-elevation of Central, Northern and Venezuelan Andes during the last 11 Ma (modified from Hoorn et al., 2010); biogeographical regions employed for ancestral area estimation analyses. The delimitation of the Chocó follows that of (Antonelli et al., 2009)]. Photos: D. Bogarín, A. Karremans, O. Pérez-Escobar.

Figure S2 Spatio-temporal macro-evolutionary dynamics of the orchid clade Cymbidieae in the American tropics (adapted from Pérez-Escobar et al., 2017a). (A) Bayesian chronogram of the Pleurothallidinae, with ancestral areas estimated at nodes using the Dispersal, Extinction and Cladogenesis and the founder speciation event models as implemented in the R package BioGeoBEARS. Branches are colour coded according to the ancestral area estimated at the parent node. The distribution of the species sampled in the phylogeny is shown as horizontal colour coded bars in front of the tree terminals. Three clades with nested migration events to the Chocó region are highlighted in grey and labelled with roman numerals (I–III). (B). Zoom-in of clades with nested migrations to the Chocó region (highlighted with pink arrows) (Inset: a: *Cycnoches lehmannii*, b: *Dichaea globosa*, c: *Euryblema andreae*, d: *Chaubardiella pubescens*, e: *Gongora* sp.). Letters at nodes represent inferred ancestral areas. (C) Maximum Likelihood Ancestral State Estimation analyses of altitude as a continuous variable of clades I-III using a Brownian motion model as implemented in the R package phytools. Branches are colour coded as a function of estimated ancestral altitude values. [Inset: mean paleo-elevation of Central, Northern and Venezuelan Andes during the last 11 Ma (modified from Hoorn et al., 2010); biogeographical regions employed for ancestral area estimation analyses. The delimitation of the Chocó follows that of (Antonelli et al., 2009)]. Photos: D. Bogarín, O. Pérez-Escobar.

## References

[B1] Antonelli A. Ariza M. Albert J. Andermann T. Azevedo J. Bacon C. (2018a). Conceptual and empirical advances in Neotropical biodiversity research. PeerJ 6, e5644. 10.7717/peerj.5644 30310740 PMC6174874

[B2] Antonelli A. Nylander J. A. A. Persson C. Sanmartín I. (2009). Tracing the impact of the Andean uplift on Neotropical plant evolution. PNAS 106, 9749–9754. 10.1073/pnas.0811421106 19470489 PMC2685738

[B3] Antonelli A. Sanmartín I. (2011a). Why are there so many plant species in the Neotropics? Taxon 60, 403–414. 10.1002/tax.602010

[B4] Antonelli A. Sanmartín I. (2011b). Mass Extinction, gradual cooling, or rapid radiation? reconstructing the spatiotemporal evolution of the ancient angiosperm genus *Hedyosmum* (Chloranthaceae) using empirical and simulated approaches. Syst. Biol. 60, 596–615. 10.1093/sysbio/syr062 21856636

[B5] Antonelli A. Zizka A. Carvalho F. A. Scharn R. Bacon C. D. Silvestro D. (2018b). Amazonia is the primary source of Neotropical biodiversity. PNAS 115, 6034–6039. 10.1073/pnas.1713819115 29760058 PMC6003360

[B6] Arditti J. Ghani A. (2000). Numerical and physical properties of orchid seeds and their biological implications. New Phyt. 145, 367–421. 10.1046/j.1469-8137.2000.00587.x 33862900

[B7] Bacon C. D. Mora A. Wagner W. L. Jaramillo C. A. (2013). Testing geological models of evolution of the Isthmus of Panama in a phylogenetic framework. Bot. J. Lin. Soc. 171, 287–300. 10.1111/j.1095-8339.2012.01281.x

[B8] Bacon C. D. Silvestro D. Jaramillo C. Smith B. T. Chakrabarty P. Antonelli A. (2015). Biological evidence supports an early and complex emergence of the Isthmus of Panama. PNAS 112, 6110–6115. 10.1073/pnas.1423853112 25918375 PMC4434730

[B9] Banerjee S. Rai S. Sarma B. Joshi S. (2012). Bacterial biofilm on water bodies of Cherrapunjee: the rainiest place on planet Earth. Adv. Microbiol. 2, 465–475. 10.4236/aim.2012.24060

[B10] Bernal R. Galeano G. (2013). *Sabinaria*, a new genus of palms (Cryosophileae, Coryphoideae, Arecaceae) from the Colombia-Panama border. Phytotaxa 144, 27–44. 10.11646/phytotaxa.144.2.1

[B11] Behling H. Hooghiemstra H. Negret A. J. (1998). Holocene history of the Chocó rain forest from Laguna Piusbi, Southern Pacific lowlands of Colombia. Quaternary Res. 50, 300–308. 10.1006/qres.1998.1998

[B12] Bogarín D. Pupulin F. Rincón R. Serracín Z. Samudio Z. (2015). An updated checklist of the Orchidaceae of Panamá. Lankesteriana 14, 135–364. 10.15517/lank.v14i3.17958

[B13] Borrero C. Pardo A. Jaramillo C. M. Osorio J. A. Cardona A. Flores A. (2012). Tectonostratigraphy of the Cenozoic Tumaco forearc basin (Colombian Pacific) and its relationship with the northern Andes orogenic build up. J. South Am. Earth Sci. 39, 75–92. 10.1016/j.jsames.2012.04.004

[B14] Canal D. Celis M. Croat T. B. Borsch T. Jones K. E. (2019). Out of Amazonia and back again: historical biogeography of the species-rich Neotropical genus *Philodendron* (Araceae). Ann. Bot. 104, 49–68. 10.3417/2018266

[B15] Cano Á. Bacon C. D. Stauffer F. W. Antonelli A. Serrano-Serrano M. L. Perret M. (2018). The roles of dispersal and mass extinction in shaping palm diversity across the Caribbean. J. Biogeo. 45, 1432–1443. 10.1111/jbi.13225

[B16] Cano A. Manrique H. F. Hoyos-Gomez S. E. Echavarria N. Upedui A. Gonzalez M. F. (2017). Palms of the Darién Gap (Colombia-Panama). Palms 61, 5–20.

[B17] Cardona A. Leon S. Jaramillo J. S. Montes C. Valencia V. Vanegas J. (2018). The Paleogene arcs of the northern Andes of Colombia and Panama: insights on plate kinematic implications from new and existing geochemical, geochronological and isotopic data. Tectonophysics 749, 88–103. 10.1016/j.tecto.2018.10.032

[B18] Christenhusz M. J. Fay M. F. Chase M. W. (2017). Plants of the world: an illustrated encyclopedia of vascular plants. London: Kew Publishing, 792 p. 10.7208/chicago/9780226536705.001.0001

[B19] Coates A. G. Collins L. S. Aubry M. P. Berggren W. A. (2004). The geology of the Darien, Panama, and the late Miocene-Pliocene collision of the Panama Arc with northwestern South America. Geol. Soc. Am. Bull. 116, 1327–1344. 10.1130/B25275.1

[B20] Copete J. C. Sánchez M. Camara-Leret M. Balslev H. (2019). Diversity of palm communities in the biogeographic Chocó and its relation with precipitation. Caldasia 41, 358–369. 10.15446/caldasia.v41n2.66576

[B21] DRYFLOR. (2016). Plant diversity patterns in Neotropical dry forests and their conservation implications. Science 353, 1383–1387. 10.1126/science.aaf5080 27708031

[B22] Duque-Caro H. (1990a). The Chocó block in the northwestern corner of South America: structural, tectonostratigraphic, and paleogeographic implications. J. South Am. Earth Sci. 3, 71–84. 10.1016/0895-9811(90)90019-W

[B23] Duque-Caro H. (1990b). Neogene stratigraphy, paleoceanography and paleobiogeography in northwest South America and evolution of the Panama Seaway. Palaeogeogr. Palaeoclimatol. Palaeoecol. 77, 203–234. 10.1016/0031-0182(90)90178-A

[B24] Eiserhardt W. L. Couvreur T. L. P. Baker W. J. (2017). Plant phylogeny as a window on the evolution of hyperdiversity in the tropical rainforest biome. New Phyt. 214, 1–15. 10.1111/nph.14516 28277624

[B25] Erkens R. H. J. Chatrou L. W. Maas J. W. van der Niet T. Savolainen V. (2007). A rapid diversification of rainforest trees (*Guatteria*; Annonaceae) following dispersal from Central into South America. Mol. Phylogenet. Evol. 44, 399–411. 10.1016/j.ympev.2007.02.017 17433720

[B26] Escobar S. Pintaud J.-C. Baslev H. Bernal R. Ramírez M. Millán B. (2018). Genetic structuring in a Neotropical palm analyzed through an Andean orogenesis-scenario. Ecol. Evol. 8, 8030–8042. 10.1002/ece3.4216 30250682 PMC6144996

[B27] Fedorov A. V. Brierley C. M. Lawrence K. T. Liu Z. Dekens P. S. Ravelo A. C. (2013). Patterns and mechanisms of early Pliocene warmth. Nature 496, 43–49. 10.1038/nature12003 23552943

[B28] Forero E. Gentry A. H. (1989). Lista anotada de las plantas del Departamento del Chocó, Colombia. Bogota: Inst. Cienc. Nat. Museo Hist. Nat. 1–142.

[B29] Galeano G. Suárez S. Balslev H. (1998). Vascular plant species count in a wet forest in the Chocó area on the Pacific coast of Colombia. Biodiv. Cons. 7, 1563–1575. 10.1023/A:1008802624275

[B30] Gentry A. H. (1982a). Neotropical floristic diversity: phytogeographical connections between Central and South America, Pleistocene climatic fluctuations, or an accident of the Andean orogeny? Ann. M. Bot. 69, 557–593. 10.2307/2399084

[B31] Gentry A. H. (1982b). “Biological diversification in the Tropics,” in Phytogeographic patterns as evidence for a Chocó refuge. Eds. Prance and G. T. (New York: Columbia University Press), 112–136.

[B32] Gentry A. H. (1986). Species richness and floristic composition of Chocó region plant communities. Caldasia 15, 71–75.

[B33] Gentry A. H. (1992). Tropical forest biodiversty: distributional patterns and their conservational significance. Oikos 63, 19–28. 10.2307/3545512

[B34] Haffer J. (1970). Geological-climatic history and zoogeographic significance of the Uraba region in northwestern Colombia. Caldasia 50, 603–636.

[B35] Hoorn C. Wesselingh F. P. (2010). Amazonia: landscape and evolution. Oxford: Wiley-Blackwell, 482 p. 10.1002/9781444306408

[B36] Hoorn C. Wesselingh F. P. Steege H. Bermudez M. A. Mora A. Sevink J. (2010). Amazonia through time: Andean uplift, climate change, landscape evolution, and biodiversity. Science 330, 927–931. 10.1126/science.1194585 21071659

[B37] Huang D. E. Goldberg E. Chou L. M. Roy K. (2017). The origin and evlution of coral species richness in a marine biodiversity hotspot. Evolution 72, 288–302. 10.1111/evo.13402 29178128

[B38] Hughes C. Eastwood R. (2006). Island radiation on a continental scale: exceptional rates of plant diversification after uplift of the Andes. PNAS 103, 10334–10339. 10.1073/pnas.0601928103 16801546 PMC1502458

[B39] Jaramillo C. (2018). “Mountains, Climate and Biodiversity,” in Evolution of the Isthmus of Panama: biological, palaeoceanographic and palaeoclimatological implications. Eds. Hoorn C. Perrigo A. Antonelli A. (London: Wiley-Blackwell), 323–338.

[B40] Jaramillo M. A. (2006). Using *Piper* species diversity to identify conservation priorities in the Chocó region of Colombia. Biodiv. Cons. 15, 1659–1712. 10.1007/s10531-004-5018-9

[B41] Kirby S. H. (2016). Active tectonic and volcanic mountain building as agents of rapid environmental changes and increased orchid diversity and long-distance orchid dispersal in the tropical Americas: opportunities and challenges. Lankesteriana 16, 243–254. 10.15517/lank.v16i2.26027

[B42] Lagomarsino L. Condamine F. L. Antonelli A. Mulch A. Davis C. C. (2016). The abiotic and biotic drivers of rapid diversification in Andean bellflowers (Campanulaceae). New Phyt. 210, 1430–1432. 10.1111/nph.13920 PMC4950005 26990796

[B43] Lellinger D. B. (1975). A phytogeographic analysis of the Chocó pteridophytes. Fern Gaz. 11, 105–114.

[B44] Lellinger D. B. de la Sota E. (1978). The phytogeography of the Pteridophytes of the Departamento del Chocó. Res. Rep. Natl. Georgr. Soc. 1969, 381–387.

[B45] León S. Cardona A. Parra M. Sobel E. R. Jaramillo J. S. Glodny J. (2018). Transition From Collisional to Subduction-Related Regimes: An Example From Neogene Panama-Nazca-South America Interactions. Tectonics 37, 119–139. 10.1002/2017TC004785

[B46] Martins A. C. Bochorny T. Pérez-Escobar O. A. Chomicki G. Monteiro S. H. N. Smidtm E. C. (2018). From tree tops to the ground: reversals to terrestrial habit in *Galenadra* orchids (Epidendroideae: Catasetinae). Mol. Phylogenet. Evol. 127, 952–960. 10.1016/j.ympev.2018.06.041 29969657

[B47] Mittermeier R. A. Turner W. R. Larsen F. W. Brooks T. M. Gascon C., (2011). “Biodiversity hotspots,” in Global biodiversity conservatrion: the critical role of hotspots. Eds. Zachos F. E. Habel J. C. (London: Springer-Verlag), 3–22. 10.1007/978-3-642-20992-5_1

[B48] Montes C. Cardona A. Jaramillo C. Pardo A. Silva J. C. Valencia V. (2015). Middle Miocene closure of the Central American Seaway. Science 348, 226–229. 10.1126/science.aaa2815 25859042

[B49] Mora M. Bernal R. Croat T. Jácome J. (2006). A phytogeographic analysis of Araceae of Cabo Corrientes (Chocó, Colombia) and comparable lowland tropical American floras. Ann. M. Bot. 93, 359–366. 10.3417/0026-6493(2006)93[359:APAOAO]2.0.CO;2

[B50] Morrone J. J. (2006). Biogeographic areas and transition zones of Latin America and the Caribbean islands based on panbiogeographic and cladistic analyses of the entomofauna. Ann. Rev. Ento. 51, 467–494. 10.1146/annurev.ento.50.071803.130447 16332220

[B51] Murren C. Ellison A. (1998). Seed dispersal characteristics of *Brassavola* nodosa (Orchidaceae). Am. J. Bot. 85, 675–680. 10.2307/2446537 21684949

[B52] Myers N. Mittermeier R. A. Mittermeier C. G. Fonseca G. A. B. Kent J. (2000). Biodiversity hotspots for conservation priorities. Nature 403, 853–858. 10.1038/35002501 10706275

[B53] Nevado B. Atchison G. W. Hughes C. E. Filatov D. A. (2016). Widespread adaptive evolution during repeated evolutionary radiations in New World lupins. Nat Commun. 7, 1–9. 10.1038/ncomms12384 PMC4979066 27498896

[B54] Pennington R. T. Quentin B. C. Richardson J. A. (2004). Introduction and synthesis: plant phylogeny and the origin of major biomes. Phil. Trans. R. Soc. Lond. B. 359, 1455–1464. 10.1098/rstb.2004.1539 15519964 PMC1693442

[B55] Pennington R. T. lavin M. Oliveira-Fliho A. (2009). Woody plant diveristy, evolution, and ecology in the tropics: perspectives from Seassonally Dry Tropical Forests. Ann. Revs. 40, 437–457. 10.1146/annurev.ecolsys.110308.120327

[B56] Pérez-Escobar O. A. (2016). Molecular phylogenetics, evolution of sexual systems andhistorical biogeography of Darwin’s favourite orchids (Catasetinae) and swan orchids (*Cycnoches* Lindl.). [disertation thesis]. [Munich]: Ludwig-Maximilians Universität.

[B57] Pérez-Escobar O. A. Chomicki G. Condamine F. L. Karremans A. P. Bogarín D. Matzke N. J. (2017a). Recent origin and rapid speciation of Neotropical orchids in the world’s richest plant biodiversity hotspot. New Phyt. 215, 891–905. 10.1111/nph.14629 PMC5575461 28631324

[B58] Pérez-Escobar O. A. Chomicki G. Condamine F. L. De Vos J. M. Martins A. C. Smidt E. C. (2017b). Multiple geographical origins of environmental sex determination enhanced the diversification of Darwin’s favourite orchids. Sci. Rep. 7, 12878. 10.1038/s41598-017-12300-y 29018291 PMC5635016

[B59] Pérez-Escobar O. A. Gottschling M. Chomicki G. Condamine F. L. Klitgård B. B. Pansarin E. (2017c). Andean mountain building did not preclude dispersal of lowland epiphytic orchids in the Neotropics. Sci. Rep. 7, 4919. 10.1038/s41598-017-04261-z 28687774 PMC5501825

[B60] Pérez-Escobar O. A. Cass S. Dodsworth S. (2018). Digest: drivers of coral diversification in a major marine biodiversity hotspot. Evolution 72, 406–408. 10.1111/evo.13419 29319173

[B61] Pirie M. D. Maas P. J. M. Wilschut R. A. Melchers-Sharrott H. Chatrou L. W. (2018). Parallel diversifications of *Cremastosperma* and *Mosannona* (Annonaceae), tropical rainforest trees tracking Neogene upheaval of South America. R. Soc. Open Sci. 5, 171561. 10.1098/rsos.171561 29410860 PMC5792937

[B62] Poveda G. Mesa A. (2000). On the existence of Lloró (the rainiest locality on Earth): enhanced ocean-land-atmosphere interaction by a low-level Jet. Geophys. Res. Lett. 27, 1675–1678. 10.1029/1999GL006091

[B63] Rangel-Chui J. O. (2011). Diversidad biótica IV: el Chocó biogeografico/Costa Pacífica. Bogota: Universidad Nacional de Colombia, Instituto de Ciencias Naturales, Conservación Internacional.

[B64] Richardson J. E. Pennington R. T. Pennington T. D. Hollingsworth P. M. (2001). Rapid diversification of a species-rich genus of Neotropical rain forest trees. Science 293, 2242–2245. 10.1126/science.1061421 11567135

[B65] Schley R. J. de la Estrella M. Pérez-Escobar O. A. Bruneau A. Barraclough T. Forest F. (2018). Is Amazonia a ‘museum’ for Neotropical trees? The evolution of the *Brownea* clade (Detarioideae, Leguminosae). MPE 126, 279–292. 10.1016/j.ympev.2018.04.029 29702213

[B66] Simon M. F. Grether R. Queiroz L. P. Skema C. Pennington R. T. Hughes C. E. (2009). Recent assembly of the Cerrado, a Neotropical plant diversity hotspot, by in situ evolution of adaptations to fire. PNAS 106, 20359–20364. 10.1073/pnas.0903410106 19918050 PMC2787167

[B67] Testo W. L. Sessa E. Barrington D. S. (2019). The rise of the Andes promoted rapid diversification in Neotropical *Phlegmariurus* (Lycopodiaceae). New Phyt. 222, 604–613. 10.1111/nph.15544 30326543

[B68] Thode V. A. Sanmartín I. Lohmann L. G. (2019). Contrasting patterns of diversification between Amazonian and Atlantic forest clades of Neotropical lianas (*Amphilophium*, Bignoniaceae) inferred from plastid genomic data. Mol. Phylogenet. Evol. 133, 92–106. 10.1016/j.ympev.2018.12.021 30584919

[B69] Thornhill A. H. Ho S. Y. W. Külheim C. Crisp M. D. (2015). Interpreting the modern distribution of Myrtaceae using a dated molecular phylogeny. MPE 93, 29–43. 10.1016/j.ympev.2015.07.007 26211451

[B70] Uribe-Convers S. Tank D. C. (2015). Shifts in diversification rates linked to biogeographic movement into new areas: an example of a recent radiation in the Andes. Am. J. Bot. 102, 1–16. 10.3732/ajb.1500229 26542843

[B71] Urreta G. M. (2005). Orquideas de la Serrania del Baudo, Chocó, Colombia. Medellin: Corporación Capitalina de Orquideología.

[B72] Vasconcelos T. N. C. Chartier M. Prenner G. Martins A. C. Schönenberger J. Wingler A. (2019). Floral uniformity through evolutionary time in a species-rich tree lineage. New Phyt. 221, 1597–1608. 10.1111/nph.15453 30284282

[B73] Vilhena D. Antonelli A. (2015). A network approach for identifying and delimiting biogeographical regions. Nat. Commun. 6, 6848. 10.1038/ncomms7848 25907961 PMC6485529

[B74] Wegner W. Wörner G. Harmon M. E. Jicha B. R. (2011). Magmatic history and evolution of the Central American land bridge in Panama since the Cretaceous times. Geol. Soc. Am. Bull. 123, 703–724. 10.1130/B30109.1

[B75] Winterton C. Richardson J. E. Hollingsworth M. Clark A. (2014). “Paleobotany and Biogeography: a Festschrift for Alan Graham in his 80th year,” in Historical biogeography of the Neotropical legume genus Dussia: the Andes, the Panama Isthmus and the Chocó. Eds. Stevens W. D. Montiel O. M. Raven P. (Missouri: Missouri Botanical Garden Press).

