# Supplementary figures and images for: The Origin and Diversification of the Hyperdiverse Flora in the Chocó Biogeographic Region
Source: Front Plant Sci. 2019 Dec 6;10:1328. doi: 10.3389/fpls.2019.01328 (PMC6910151; doi:10.3389/fpls.2019.01328)

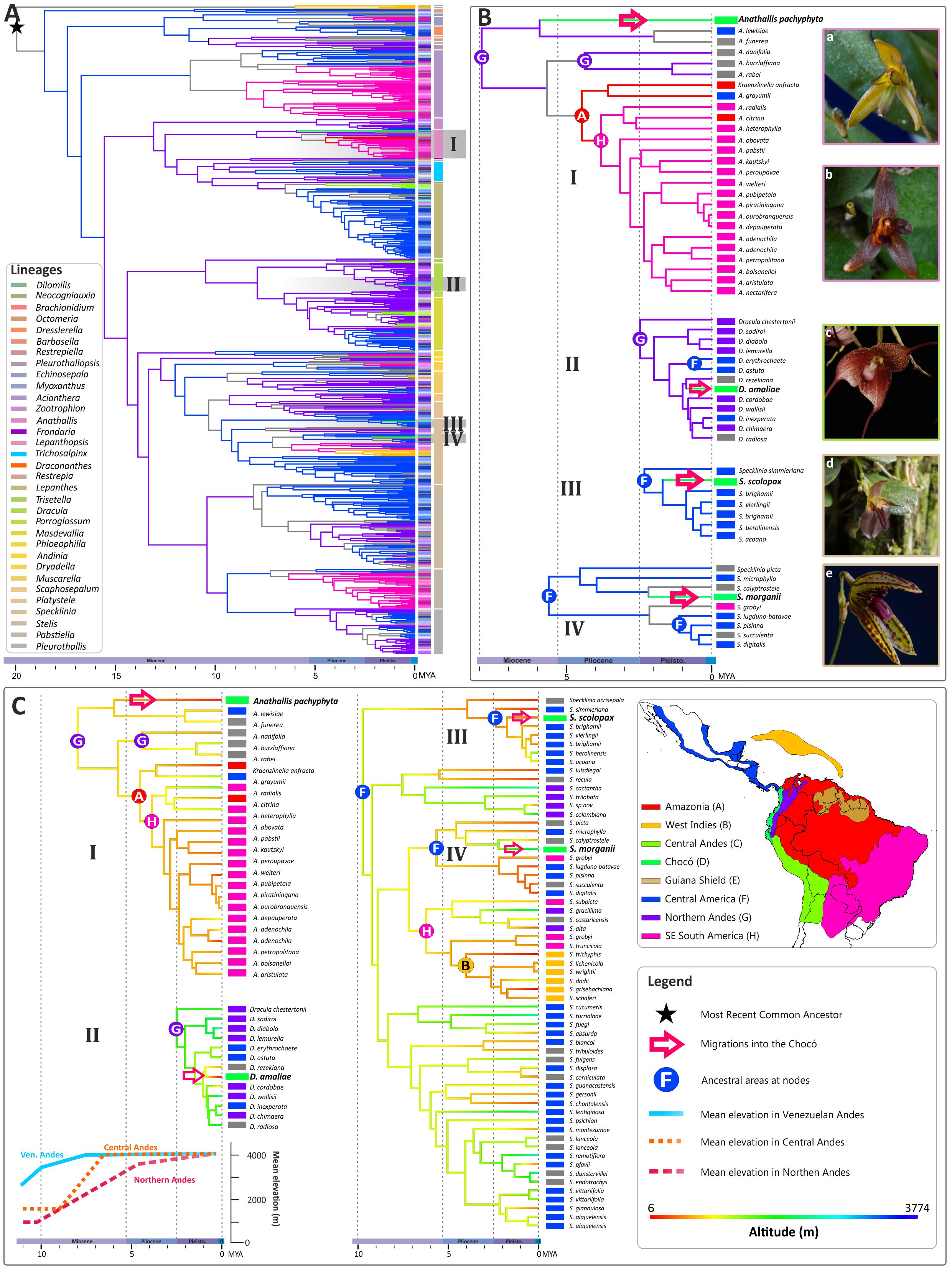

Supplement: Figure S1 — Spatio-temporal macro-evolutionary dynamics of the orchid clade Pleurothallidinae in the American tropics (adapted from Pérez-Escobar et al., 2017a). (A) Bayesian chronogram of the Pleurothallidinae, with ancestral areas estimated at nodes using the Dispersal, Extinction and Cladogenesis and the founder speciation event models as implemented in the R package BioGeoBEARS. Branches are colour coded according to the ancestral area estimated at the parent node. The distribution of the species sampled in the phylogeny is shown as horizontal colour coded bars in front of the tree terminals. Four clades with nested migration events to the Chocó region are highlighted in grey and labelled with roman numerals (I–IV). (B). Zoom-in on clades with nested migrations to the Chocó region (highlighted with pink arrows) (Inset: a: Anathallis funerea, b: Anathallis lewisiae, c: Dracula sp., d: Specklinia pereziana, e: Specklinia berolinensis). Letters at nodes represent inferred ancestral areas. (C) Maximum Likelihood Ancestral State Estimation analyses of altitude as a continuous variable of clades I-IV using a Brownian motion model as implemented in the R package phytools. Branches are colour coded as a function of estimated ancestral altitude values. [Inset: mean paleo-elevation of Central, Northern and Venezuelan Andes during the last 11 Ma (modified from Hoorn et al., 2010); biogeographical regions employed for ancestral area estimation analyses. The delimitation of the Chocó follows that of (Antonelli et al., 2009)]. Photos: D. Bogarín, A. Karremans, O. Pérez-Escobar. [file Image_1.jpeg]

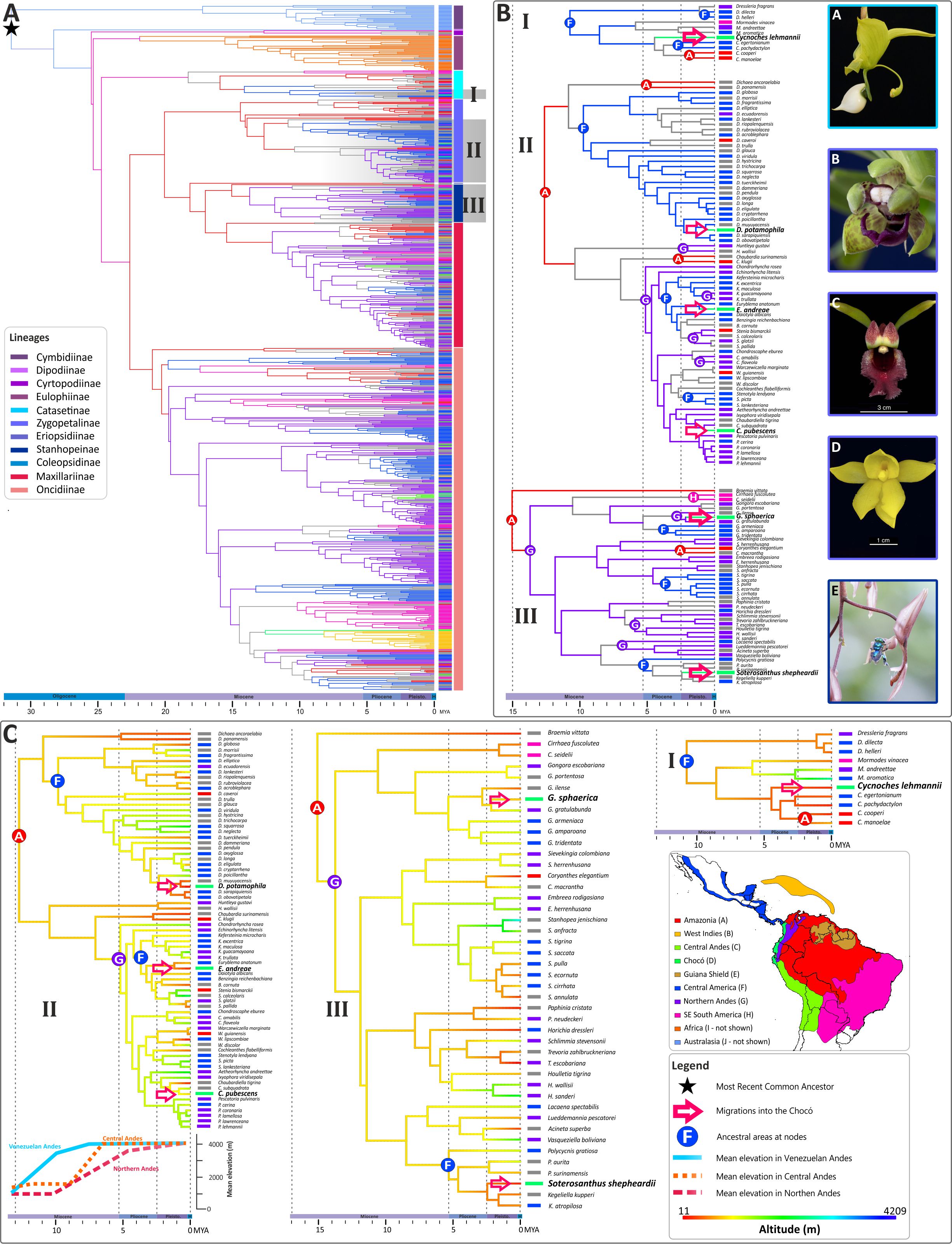

Supplement: Figure S2 — Spatio-temporal macro-evolutionary dynamics of the orchid clade Cymbidieae in the American tropics (adapted from Pérez-Escobar et al., 2017a). (A) Bayesian chronogram of the Pleurothallidinae, with ancestral areas estimated at nodes using the Dispersal, Extinction and Cladogenesis and the founder speciation event models as implemented in the R package BioGeoBEARS. Branches are colour coded according to the ancestral area estimated at the parent node. The distribution of the species sampled in the phylogeny is shown as horizontal colour coded bars in front of the tree terminals. Three clades with nested migration events to the Chocó region are highlighted in grey and labelled with roman numerals (I–III). (B). Zoom-in of clades with nested migrations to the Chocó region (highlighted with pink arrows) (Inset: a: Cycnoches lehmannii, b: Dichaea globosa, c: Euryblema andreae, d: Chaubardiella pubescens, e: Gongora sp.). Letters at nodes represent inferred ancestral areas. (C) Maximum Likelihood Ancestral State Estimation analyses of altitude as a continuous variable of clades I-III using a Brownian motion model as implemented in the R package phytools. Branches are colour coded as a function of estimated ancestral altitude values. [Inset: mean paleo-elevation of Central, Northern and Venezuelan Andes during the last 11 Ma (modified from Hoorn et al., 2010); biogeographical regions employed for ancestral area estimation analyses. The delimitation of the Chocó follows that of (Antonelli et al., 2009)]. Photos: D. Bogarín, O. Pérez-Escobar. [file Image_2.jpeg]
